# Tribological Reliability of Polymer Composites for Skin-Interfaced Bioelectronics: Wear-to-Failure Pathways and Testing Framework

**DOI:** 10.3390/polym18192404

**Published:** 2026-10-01

**Authors:** Fahad Alshabouna, Majed Majrashi, Aliyah Algethami, Meshal Aljohani, Hamad Alromaih, Nasser Alqahtani, Obaid A. Alharbi

**Affiliations:** 1 Institute of Bioengineering, King Abdulaziz City for Science and Technology (KACST), Riyadh 11442, Saudi Arabia; asalgethami@kacst.gov.sa; 2 Institute of Hydrogen Technologies, King Abdulaziz City for Science and Technology (KACST), Riyadh 11442, Saudi Arabia; maljuhni@kacst.gov.sa; 3 Institute of Water Desalination Technologies, King Abdulaziz City for Science and Technology (KACST), Riyadh 11442, Saudi Arabia; halrumayh@kacst.gov.sa; 4 Institute of Waste Management and Recycling Technologies, King Abdulaziz City for Science and Technology (KACST), Riyadh 11442, Saudi Arabia; nalqahtani@kacst.gov.sa; 5 Institute of Water Management and Treatment Technologies, King Abdulaziz City for Science and Technology (KACST), Riyadh 11442, Saudi Arabia; oaharbi@kacst.edu.sa

**Keywords:** wearable bioelectronics, polymer composites, tribology, skin-device interface, mechanical durability, stretchable electronics

## Abstract

Skin-interfaced bioelectronics rely on soft polymer composites to provide electrical functionality while maintaining the compliance and deformability needed for prolonged contact with the human body. Mechanical durability is commonly demonstrated through stretchability, cyclic deformation and retention of electrical performance under strain, but these measures do not capture contact-induced damage during actual wear. Skin movement, clothing contact and repeated use introduce friction, sliding and abrasion that can produce surface wear, cracking, delamination, conductive-network disruption and instability at the skin-device interface. This review considers tribological reliability as a distinct dimension of wearable durability, linking polymer-composite architecture and interfacial design to the development of wear and its progression into electrical degradation and loss of device function. Evidence across electrophysiological, pressure, strain and biochemical devices shows that material survival, electrical continuity and functional performance do not necessarily deteriorate together, making cycle count or retained conductivity alone insufficient indicators of durability. We propose an application-specific framework that evaluates representative wear conditions together with controlled contact and environmental parameters, morphological damage, electrical stability and preservation of sensing function. Future claims of tribological reliability should therefore be based on whether the required device function is retained under a representative and sufficiently defined wear exposure.

## 1. Introduction—Beyond Stretchability: The Reliability Gap in Skin-Interfaced Bioelectronics

Soft polymer composites have become central to skin-interfaced bioelectronics because they can combine electrical functionality with the compliance, deformability and intimate contact required at the body surface [1,2,3,4,5,6,7,8]. Because skin undergoes large and repeated deformation during daily activity, the development of these systems has understandably emphasized whether conductive materials can deform with the body without losing electrical continuity [9,10,11,12]. Stretchability, cyclic deformation and retention of electrical performance under mechanical loading have therefore become common measures of mechanical durability, while adhesion and conformability are frequently used to assess mechanical compatibility with the skin [4,6,8,9,11,12,13,14]. These priorities are reflected in recent developments in soft bioelectronics, where material and device design increasingly combines intrinsically or structurally stretchable conductors, compliant polymer matrices and interface-engineering strategies to reduce mechanical mismatch while maintaining electrical performance and stable integration with the body [15,16].

These measurements are useful, but they address only one part of wearable reliability. They establish whether a device can remain functional while accommodating deformation, whereas actual use also exposes the system to repeated contact and tangential loading that are not reproduced by deformation-based mechanical tests alone [17,18]. The ability to withstand stretching, bending or other forms of deformation therefore does not necessarily indicate the ability to tolerate the mechanical interactions associated with prolonged wear. Stretchability and related deformation-based measures should consequently be regarded as important indicators of mechanical reliability, but not sufficient representations of the conditions encountered during actual use. This distinction is particularly relevant to polymer composites, in which load transfer through the polymer matrix, conductive network and material interfaces influences the development of contact-induced damage [17,18].

This gap is only partly addressed by the existing literature. Reviews of flexible and stretchable electronics largely focus on deformation, fatigue and fracture, while biointerface studies emphasize conformability, adhesion and stable coupling to the body [13,19,20]. Recent reviews of soft bioelectronic interfaces further emphasize the importance of matching the mechanical characteristics of electronic materials with biological tissues and engineering interfaces capable of maintaining intimate and stable contact during use [15,21]. Conductive textiles reviews also discuss durability, including washing and wear, but do not trace tribological exposure through material degradation to functional failure [22]. Broader assessments of electronic textiles similarly identify durability as a central challenge alongside material selection, fabrication, integration and long-term practical use [23]. Application-specific reviews examine robustness under environmental stresses in systems such as superhydrophobic strain sensors and flexible pressure sensors exposed to sweat and moisture [24,25]. Together, these bodies of literature approach wearable reliability from different perspectives, including electromechanical deformation, biointerface stability, textile durability and environmental resistance. These perspectives broaden the discussion of wearable reliability, but do not treat tribological contact as a cross-modal problem linking repeated friction and wear with material degradation, electrical deterioration and loss of bioelectronic function. Motion-artifact research addresses another related problem: transient disruption of recorded signals during movement [26]. Dedicated reviews of motion-artifact management further examine the origins and mitigation of these transient disturbances at soft bioelectronic interfaces [27]. Tribological reliability instead addresses the progressive material and interfacial deterioration caused by repeated contact, rather than temporary disturbance during motion. Accounting for progressive contact-induced damage therefore extends existing mechanical and biointerface assessments rather than replacing them.

Focusing on tribological reliability shifts the emphasis from deformation tolerance to the mechanisms by which repeated contact during real wear produces material damage, the role of polymer-composite design in mitigating that damage, and the progression into electrical or bioelectronic failure. This review critically examines these pathways and the evidence required to establish tribological reliability in skin-interfaced bioelectronics. It then proposes an application-specific testing framework linking representative wear exposure to material, electrical and functional outcomes.

## 2. Methods

During preparation of the manuscript, the authors used ChatGPT (OpenAI, GPT-5.5 and GPT-5.6) for language refinement, manuscript organization and the initial conceptual development of graphical content. All AI-assisted output was reviewed and edited by the authors. All figures were created and finalized by the authors using Adobe Illustrator 2020.

## 3. Tribological Interactions and Wear-Induced Damage in Skin-Interfaced Bioelectronics

Conformal attachment does not eliminate tribological loading in skin-interfaced bioelectronics. During movement, the skin and device deform differently, generating local microslip and shear even when adhesion is maintained [26]. At the same time, clothing can repeatedly rub exposed device surfaces, while conductive layers, substrates, encapsulants and adhesives can experience relative motion internally because of differences in stiffness and deformation response [17,18,28]. Friction during wear therefore develops at the skin-device interface, the device-environment interface and internal material-material interfaces. It is not necessarily evidence of poor attachment, but an unavoidable consequence of coupling a multilayer device to a continuously deforming biological surface.

The presence of friction, however, is not itself failure. Tribological damage begins when repeated contact is no longer accommodated reversibly and instead produces permanent material or interfacial change. This can manifest as surface material loss, cracking, conductor detachment, delamination and disruption or exposure of conductive networks [17,18,28]. The relevant reliability question is therefore not whether friction occurs, but when repeated contact begins to produce cumulative and irreversible wear.

**Figure 1:**
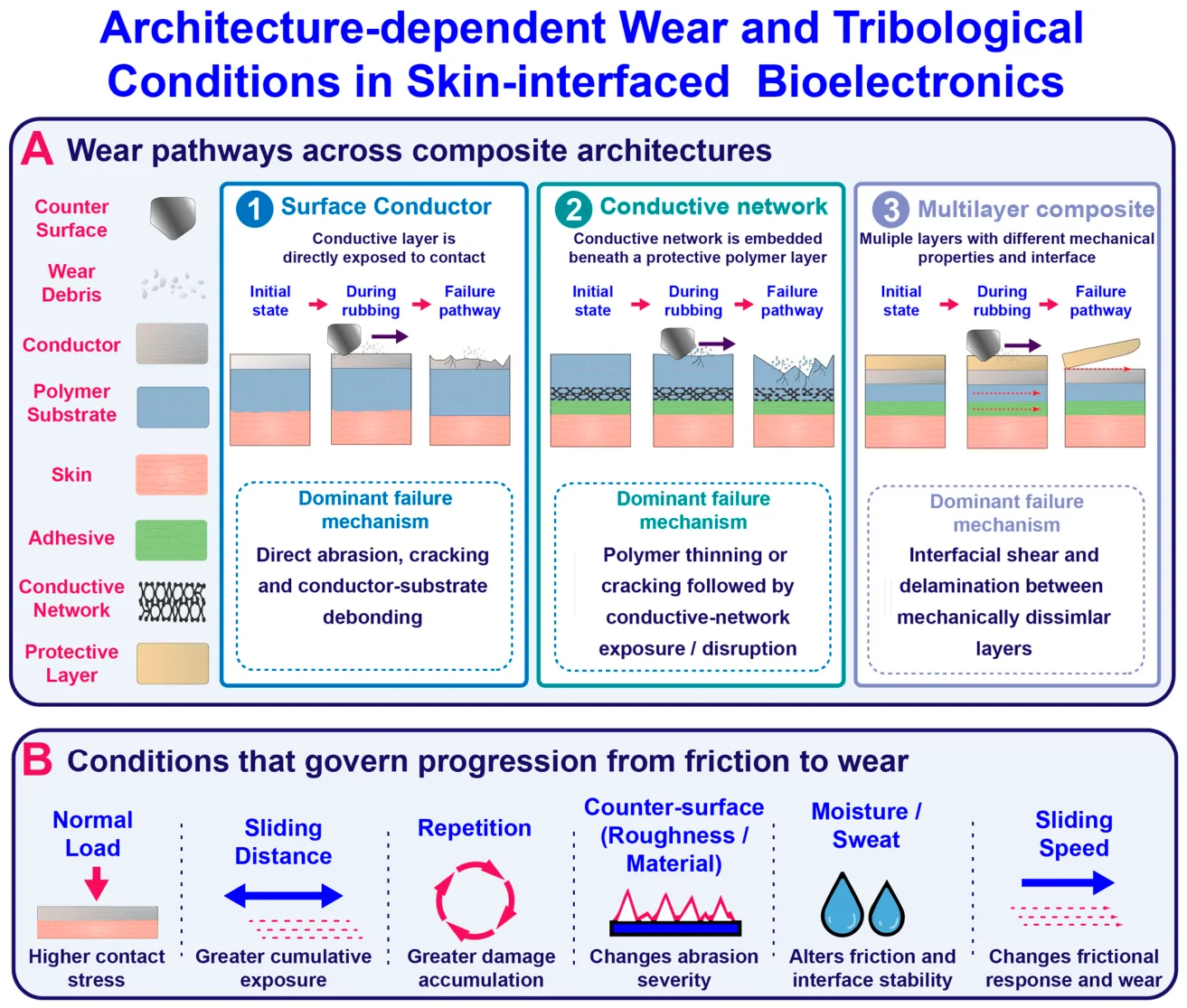
(**A**) Three archetypal load-transfer configurations in skin-interfaced bioelectronics and their dominant tribological wear pathways: surface conductors, embedded or distributed conductive networks, and multilayer composites. (**B**) Contact and environmental conditions governing wear severity. Colors distinguish the architectural configurations and material components identified in the figure. Arrows indicate rubbing, relative motion or progression of damage, and dashed outlines indicate grouped failure mechanisms.

From a tribological standpoint, the most informative architectural distinction is the mechanical load path between the contacting surface and the electrically active region. Contact can reach the conductive phase directly, through a surrounding polymer matrix, or across discrete material layers and interfaces (Figure 1A). These three load-transfer configurations provide an engineering basis for comparing tribological failure pathways, although individual devices may combine elements of more than one configuration. For devices combining these configurations, the progression of damage should be traced from the contacting surface toward the electrically active region. For example, abrasion may initially remove a protective surface layer without impairing electrical performance, whereas subsequent exposure of an embedded network or delamination at a buried interface may compromise conduction. The dominant failure pathway should therefore be identified from the damage mechanism associated with electrical or functional failure, rather than from the first visible defect or the nominal device architecture. Where several mechanisms contribute simultaneously and cannot be distinguished experimentally, they should be reported as coupled failure pathways. Surface conductors are directly exposed to rubbing and are therefore vulnerable to material removal and conductor-substrate debonding. In embedded or distributed conductive networks, the surrounding polymer initially shields the conductive phase, but progressive thinning, cracking or local damage can eventually expose or disrupt the network. Multilayer composites introduce a different failure pathway because repeated tangential loading can concentrate at interfaces and promote delamination between mechanically dissimilar layers [17,18,28].

The development of these damage pathways depends on the molecular structure and mechanical response of the polymer matrix. Chemical crosslinks constrain chain motion and contribute to elastic recovery, whereas physical junctions and chain entanglements influence stress redistribution, relaxation and resistance to crack growth [29]. Increasing crosslink density may restrict deformation under contact loading, but it can also limit chain extensibility and alter the energy dissipated during crack propagation. Consequently, higher stiffness does not necessarily imply greater resistance to progressive wear [29,30]. During repeated sliding, the viscoelastic response of the matrix also depends on the timescale of contact relative to polymer relaxation: deformation that has not recovered before subsequent contact may modify local contact geometry and stress distribution [31]. These properties influence the failure pathways differently across composite architectures. In embedded networks, matrix cracking or material removal can expose conductive fillers and disrupt their electrical connections [18,32], with the resulting resistance change depending on the connectivity of the remaining network. In multilayer structures, the ability to resist delamination depends on the resistance of the bonded interface to crack initiation and propagation under repeated shear [17,18]. Polymer stiffness, fracture resistance and interfacial adhesion must therefore be distinguished rather than treated as interchangeable measures of tribological durability.

Characterization methods should be selected according to the proposed wear mechanism. Rheological measurements and dynamic mechanical analysis can characterize polymer relaxation and viscoelastic response over relevant loading timescales, while X-ray scattering may reveal changes in chain orientation, crystallinity or filler organization where these structural features are relevant to the composite. Such measurements should be combined with examination of the worn surface and, where delamination is suspected, measurements of interfacial adhesion or fracture resistance. Importantly, bulk viscoelastic energy dissipation does not directly quantify frictional energy loss or material removal at the contacting surface. The relationship between polymer properties and tribological reliability should therefore be established through measurements of the actual damage produced under defined contact conditions.

The mitigation strategies reported in the literature reflect these different load-transfer pathways. Cao et al. [17] demonstrated the importance of conductor-substrate adhesion in surface architectures. Diffusion of waterborne polyurethane into the gold layer and hydrogen bonding around gold grains strengthened the bonded interface, reducing conductor detachment during repeated rubbing and helping preserve electrical continuity. In multilayer composites, Wang et al. [18] used a silver-particle/polymer surface layer to withstand abrasion while an underlying liquid-metal-particle/polymer layer maintained conductive pathways under large deformation. The shared polymer matrix also promoted interlayer adhesion, limiting separation between the wear-bearing and electrically functional layers.

These findings illustrate complementary strategies based on strengthening vulnerable interfaces and separating wear-bearing from electrically conductive functions. Chen et al. [32] embedded a flash-graphene assembly within a mechanically robust fibrous framework that maintained electrical stability under repeated contact-friction loading, while Cao et al. [33] used a protective fluoropolymer barrier to improve the mechanical stability of liquid-metal/polymer electronic textiles. These approaches point to three transferable design principles: strengthening interfaces, shielding or mechanically supporting the conductive phase, and separating wear-bearing from electrically functional components.

These strategies must also preserve the mechanical and sensing requirements of the device. Strengthening an interface through localized bonding can reduce conductor detachment without necessarily increasing the stiffness of the entire composite, as illustrated by Cao et al. [17]. By contrast, adding a protective layer or reinforcing the bulk matrix may increase thickness or alter conformability, depending on the material and geometry. The location of protection is particularly important: encapsulation can shield conductive interconnects from rubbing, but a barrier placed over an active electrode may alter electrical coupling to the skin, while a barrier over a biochemical sensing region may restrict analyte access. Separating wear-bearing and conductive functions also introduces an additional interface whose integrity must be maintained under repeated shear. These trade-offs are design-dependent rather than unavoidable; the ultrathin bilayer reported by Wang et al. [18], for example, combined abrasion resistance with electrophysiological recording. Tribological protection should therefore be evaluated together with the functional requirements of the intended sensing modality.

The severity of wear is further modified by the conditions of contact (Figure 1B). Skin friction changes with hydration, normal load and counter-surface properties [34,35], while sweat and humidity can also alter adhesion and stability at soft skin-device interfaces [6,11]. Moisture may therefore increase friction at one interface while softening, lubricating or weakening another, making its effect strongly material-dependent. In water-sensitive polymer matrices, moisture uptake may cause swelling or plasticization, changing modulus, viscoelastic recovery and surface conformity. These changes can modify the real contact area and interfacial shear resistance during sliding, thereby altering friction [36]. They can also change how tangential loading is transferred through the matrix to embedded conductive networks or bonded layers. Differential swelling between adjacent layers may further increase interfacial stresses [37], potentially facilitating separation when repeated shear is applied. Moisture therefore affects wear not only through changes in friction at the contacting surface, but also through changes in the mechanical response and interfacial integrity of the composite.

Environmental exposure should be selected according to the intended application rather than imposed as a universal humidity or sweat protocol. For example, a skin-contact electrode intended for prolonged exercise may require combined sweat exposure and repeated skin-device shear, whereas a protected interconnect primarily exposed to clothing may require fabric rubbing under representative humidity conditions. Moisture exposure can also change electrode-skin impedance independently of demonstrated wear [38]. Comparing moisture-only exposure with exposure to the same moisture conditions plus rubbing can help distinguish moisture-induced electrical changes from degradation caused by repeated contact. Humidity or sweat exposure and mechanical contact conditions should therefore be specified separately, even when they are applied simultaneously.

Loading conditions provide a second source of variation. Normal load, sliding speed, sliding distance and repetition define the magnitude and cumulative duration of tribological exposure and can substantially alter frictional response and wear mechanisms [39]. Together with counter-surface roughness, these parameters influence the transition from mild rubbing to progressive abrasion. Cao et al. [17] demonstrated the importance of the counter-surface directly, showing that the same conductor withstood progressively fewer rubbing cycles when tested against artificial skin, woven fabric and abrasive paper as surface roughness increased.

The same tribological exposure can therefore produce different damage pathways depending on material architecture and contact conditions. The significance of this damage ultimately depends on whether it remains a material-level change or propagates into electrical instability and loss of bioelectronic function.

## 4. From Tribological Degradation to Electrical and Functional Failure

Tribological degradation can compromise electrical performance through damage to conductive pathways and electrical interfaces. Conductive-pathway damage can increase resistance and eventually cause loss of continuity, whereas degradation of the skin-device interface can destabilize contact impedance and reduce signal quality even while the conductor itself remains electrically continuous [17,18]. These changes are not necessarily abrupt. Quantitative within-study comparisons further show how preparation choices translate into tribological performance. Cao et al. [17] reported an Au-WPU interfacial peel strength of 1017.6 N m^−1^, which increased to 1243.4 N m^−1^ with the rough microporous polyurethane configuration, approximately 100 and 4 times the values obtained with Au-PDMS and Au-SEBS, respectively. The corresponding Au-RPU electrode retained conductivity for 1022 ± 76 friction cycles at 130 kPa, demonstrating a quantitative link between interfacial preparation, resulting adhesion and resistance to rubbing-induced electrical failure.

Wang et al. [18] similarly demonstrated the effect of preparation and architectural design. Under the same RCA abrasion test with a 175 g load, the common-matrix AgPs/SEBS-LMPs/SEBS bilayer remained conductive for more than 1500 s, compared with 32 s for the LMPs/SEBS monolayer and 403 s for the AgPs/SEBS monolayer. When the bilayer was instead prepared with a heterogeneous PDMS-SEBS interface, its conductive lifetime decreased to 330 s. These comparisons quantitatively show how matrix compatibility and layer design influence resistance to tribological failure. By contrast, Chen et al. [32] monitored resistance during repeated contact-friction loading and observed minimal electrical change in an armor-protected flash-graphene electrode. Together, these studies show that tribological exposure can produce either progressive electrical degradation or sustained electrical stability, depending strongly on the protection and mechanical coupling of the conductive phase (Table 1).

Studies of electrophysiological monitoring provide direct examples of how tribological exposure can affect electrical properties and recording performance. Cao et al. [17] compared anti-friction Au electrodes supported on rough microporous waterborne polyurethane (RPU) with Au-PDMS controls. After repeated rubbing, the weakly bonded Au-PDMS electrodes showed deterioration in electrocardiographic (ECG) and electroencephalographic (EEG) recordings, while the Au-RPU electrodes maintained signal quality. Electromyographic (EMG) recordings showed a similar contrast, with Au-RPU electrodes maintaining a largely stable signal-to-noise ratio (SNR) and low skin-electrode impedance after rubbing [17]. Wang et al. [18] provided an even more explicit comparison before and after abrasion. Their bilayer electrodes showed substantially smaller deterioration in ECG and EMG SNR than the corresponding monolayer structures and also maintained low skin-electrode impedance after extended abrasion [18]. Chen et al. [32] broadened this evidence using an armor-protected flash-graphene electrode that maintained electrical stability under repeated contact-friction loading and was subsequently demonstrated for ECG and EMG recording. These studies extend tribological evaluation beyond electrical survival to electrophysiological performance.

A broader group of studies combines wear-related durability testing with demonstrations of bioelectronic function but does not follow the degradation pathway as completely. Ding et al. [40] combined substantial abrasion testing of skin-deposited liquid-metal electrodes with ECG and EMG demonstrations, but did not systematically track signal quality as wear accumulated toward failure. Jeong et al. [41] likewise combined repetitive sliding tests with ECG and EMG recording using consolidated Au nanosheet electrodes, but durability and biosignal performance were largely demonstrated in parallel rather than followed as a continuous failure process. Similar evidence has been reported for textile-based wearable bioelectronics. Fabric electrodes based on poly(3,4-ethylenedioxythiophene):poly(styrene sulfonate) (PEDOT:PSS)/MXene composites retained electrical stability after abrasion and washing and were subsequently shown to acquire ECG, EMG and EEG signals comparable with commercial electrodes [42]. Grooved silver-coated textile electrodes have similarly retained ECG functionality after repeated abrasion and laundering [43]. Cao et al. [33] further combined finger-friction, tape-peeling and washing durability with reusable epidermal biopotential recording in liquid-metal/polymer electronic textiles. These studies strengthen the evidence that wear-resistant architectures can preserve bioelectronic operation, but they provide less information about how progressive wear quantitatively translates into loss of measurement quality.

Tracking electrical and functional performance throughout cumulative wear presents different experimental challenges. Electrical resistance can be measured during repeated rubbing when the test configuration maintains reliable connections without changing the imposed contact conditions. Functional monitoring is more demanding because the measurement interface must remain accessible while the device is subjected to controlled friction. In electrophysiological electrodes, movement during rubbing can generate transient signal disturbances that obscure persistent deterioration in recording quality, while changes in skin contact and physiological signal amplitude complicate comparisons between wear intervals. For pressure and strain sensors, the abrasion apparatus can impose deformation or loading unrelated to the calibrated sensing input. In biochemical sensors, repeated measurements additionally require controlled analyte delivery and stable measurement conditions so that changes in analytical response can be distinguished from sample variation or sensor drift. These constraints can be addressed by combining electrical measurements during wear with functional measurements at predefined wear intervals under consistent measurement conditions. Such an approach resolves the progression of damage more clearly than a single post-wear measurement, although it does not provide uninterrupted functional monitoring during rubbing.

The distinction between electrical continuity, visible damage and sensor function becomes particularly clear outside electrophysiological electrodes. Fang et al. [44] evaluated pressure-sensing performance after repeated rubbing against 1000-mesh sandpaper and found that the capacitance-pressure response and sensitivity remained largely unchanged after 100 cycles. This provides direct evidence of preserved sensing performance after wear rather than merely retained electrical continuity. Jiang et al. [45] observed wear of the encapsulation layer, while the underlying SEBS-based composite and conductive fabric remained intact and pressure-sensing performance was largely preserved. Lee et al. [46] further demonstrated that a friction-resistant nanomesh strain sensor could continue detecting fingertip deformation under severe frictional conditions. Similar effects are seen in biochemical sensing. A core-sheath textile sweat sensor designed to shield internal electrodes from rubbing maintained pH and ion-sensing responses after simulated skin-fabric friction and prolonged on-body wear, although measurable potential shifts remained after repeated loading [47]. The relevant functional endpoint therefore depends on the sensing modality: signal quality and impedance for electrophysiological electrodes, sensitivity and response stability for physical sensors, and analytical stability for biochemical sensors.

**Table 1:** Representative studies linking tribological exposure to electrical and functional outcomes in skin-interfaced bioelectronic devices.

Study	Polymer Matrix and Device Architecture	Tribological Exposure	Electrical Assessment	Functional Assessment	Tribological Interpretation
Cao et al. [17]	Au-WPU and Au-RPU epidermal electrodes, with Au-PDMS and Au-SEBS controls	Cyclic on-skin rubbing under approximately 65 kPa; additional tests evaluated artificial skin, woven fabric and abrasive paper under defined loading.	Au-WPU sheet resistance increased from 8.7 to 35.7 Ω sq^−1^ after 1000 on-skin rubbing cycles; Au-PDMS and Au-SEBS failed after 5 and 70 cycles in the same comparison	ECG and EEG recordings remained stable after cyclic rubbing; Au-RPU electrodes maintained high EMG SNR and low skin-electrode impedance after rubbing	Demonstrates progressive electrical degradation and shows that interfacial design and counter-surface strongly influence wear resistance and electrophysiological performance. The Au-RPU interface exhibited a peel strength of up to 1243.4 N m^−1^.
Wang et al. [18]	Bilayer conductor comprising abrasion-resistant AgPs/SEBS and conductive LMPs/SEBS layers (total thickness: approximately 13.3 µm).	Standard RCA abrasion under a 175 g load; electrophysiological evaluation after 500 abrasion cycles; skin-electrode impedance after 2000 cycles	Bilayer resistance remained relatively stable for >1500 s of RCA abrasion before increasing toward failure; LMPs/SEBS and AgPs/SEBS monolayers lost conductivity after 32 and 403 s	ECG SNR changed from 20.49 to 18.75 dB and EMG SNR from 33.39 to 27.86 dB after 500 abrasion cycles; low skin-electrode impedance was retained after 2000 cycles	Provides one of the clearest links between abrasion, electrical evolution and preservation of electrophysiological performance
Chen et al. [32]	Armor-protected flash-graphene assembly embedded within polypropylene melt-blown nonwoven fabric	10,000 contact-friction cycles under a 10 N force at 4 Hz	Initial sheet resistance: 125.2 ± 4.7 Ω sq^−1^. Electrical resistance was monitored during friction and showed almost no change over 10,000 cycles	ECG and EMG monitoring demonstrated using the on-skin electrodes	Shows that physically shielding the conductive network can strongly suppress friction-induced electrical degradation, although progressive functional deterioration toward failure was not mapped
Ding et al. [40]	Polyvinyl-alcohol-modified liquid-metal particles deposited directly onto the skin, without a separate polymer substrate or encapsulating layer.	Standard abrasion testing using 175 g loaded paper tape over approximately 1600 cm sliding distance	Conductivity was retained under substantial abrasion	ECG and EMG recording demonstrated	Demonstrates electrical wear resistance together with electrophysiological functionality, although progressive functional deterioration toward failure was not mapped
Xu et al. [42]	PEDOT:PSS/MXene fabric epidermal electrode on PDA-modified cotton textile	Abrasion and washing durability tests	Electrical and signal stability were retained after washing and abrasion testing	ECG, EMG and EEG recordings were comparable with commercial Ag/AgCl electrodes	Combines wear-related durability evaluation with high-quality electrophysiological recording, but does not map progressive degradation toward functional failure
Fang et al. [44]	Microstructured PEDOT:PSS/waterborne polyurethane electrode coated with gold, integrated with a polyurethane-based ion-selective layer for pressure and potassium-ion sensing.	Rubbing against 1000-mesh sandpaper for 100 cycles	No separate conductivity endpoint was reported; durability was assessed from the capacitance-pressure response	Initial pressure sensitivity was 25.2 pF kPa^−1^ over approximately 0–20 kPa. Capacitance-pressure response and sensitivity remained largely unchanged after rubbing	Direct post-wear functional testing shows that tribological reliability can be demonstrated through preservation of sensing performance rather than electrical continuity alone
Jiang et al. [45]	SEBS-based structure-gradient fiber-mat triboelectric pressure-sensing textile. Young modulus of the tested constituent fiber mats: 0.18-0.32 MPa, depending on carbon-nanotube content.	10,000 abrasion cycles	No separate resistance or impedance failure endpoint was reported; the SGFM and conductive fabric remained physically intact	Pressure-sensing performance was well maintained despite wear of the encapsulation layer	Demonstrates that visible material wear does not necessarily translate into immediate functional failure
Luo et al. [47]	Multilayer core-sheath yarn sensor incorporating a protective thermoplastic polyurethane layer, a polyaniline/polyurethane pH-sensing sheath, moisture-transport yarns and internal ion-selective electrodes.	Simulated skin-fabric cyclic rubbing under different loads and 36 h of on-body natural wear	Potential stability was quantified after rubbing and prolonged wear	pH, Na^+^ and K^+^ sensing remained operational, although measurable potential shifts occurred. Potential changes after rubbing were 20.4% (1000 cycles, 20 g), 17.6% (200 cycles, 100 g) and 13.6% (100 cycles, 500 g), relative to the initial response.	Shows that biochemical sensing can remain functional despite wear-induced changes in analytical response

Abbreviations: AgPs, silver particles; Au, gold; ECG, electrocardiography; EEG, electroencephalography; EMG, electromyography; LMPs, liquid metal particles; PDA, polydopamine; PDMS, polydimethylsiloxane; PEDOT:PSS, poly(3,4-ethylenedioxythiophene):poly(styrene sulfonate); RCA, Radio Corporation of America abrasion test; RPU, rough microporous waterborne polyurethane; SEBS, styrene-ethylene-butylene-styrene; SGFM, structure-gradient fiber mat; SNR, signal-to-noise ratio; WPU, waterborne polyurethane.

Physical damage, electrical degradation and functional performance need not evolve together. Surface characterization should therefore be interpreted alongside electrical and functional measurements rather than used as an independent indicator of failure. Morphological analysis can establish whether wear remains confined to a protective layer, reaches the active material or disrupts the conductive architecture, but its importance ultimately depends on the resulting effect on device performance.

Bioelectronic failure should consequently not be defined solely as loss of conductivity, nor should continued signal detection be taken as evidence that sensing performance is fully preserved. For electrophysiological monitoring, deterioration of interface stability can increase or destabilize skin-electrode impedance even while the conductor itself remains continuous, thereby reducing signal fidelity without producing an electrical open circuit [17,18,48]. Li et al. [2] provide a related example under mechanical disturbance: vigorous shaking caused detachment of a commercial gel electrode and reduced the surface EMG SNR from 33.88 to 9.07 dB, whereas a strongly integrated conductive biogel maintained stable recording. In electrochemical sensors, electrical continuity may be preserved while wear-related loading shifts the analytical response [47]. The relevant failure criterion is therefore modality-dependent: physical survival, electrical continuity, signal detectability and preservation of measurement performance are not interchangeable.

Tribological failure criteria should be defined by the intended function of the device before wear testing. Material characterization identifies the location and progression of damage, while electrical measurements establish whether conductive pathways and interfaces retain their required properties. Functional assessment determines whether the device continues to meet the performance requirements of its sensing modality. These measurements should be interpreted together rather than assigned a universal ranking: visible wear does not establish functional failure when the required measurement performance is preserved, and retained conductivity does not establish reliability when sensing performance has deteriorated beyond its predefined acceptance limits.

Transient motion artifacts and cumulative wear-induced degradation can be distinguished by separating measurements obtained during movement from those obtained under controlled, stationary conditions before and after defined wear intervals. Signal fluctuations that occur during rubbing but recover when movement stops should be distinguished from persistent changes in recording quality or electrode-skin impedance that accumulate with repeated contact. To establish whether persistent deterioration is attributable to tribological damage, the comparison should include an unabraded control electrode subjected to equivalent movement and measurement conditions, together with examination of the worn electrode and its interfaces. Electrode placement, contact pressure and skin hydration should be controlled as far as practicable because changes in these conditions can alter signal quality independently of material wear. A progressive decline in stationary recording performance accompanied by measurable interfacial or conductive damage provides stronger evidence of wear-induced failure than signal fluctuations observed during movement alone.

Across studies evaluating tribological durability, the evidence differs in how directly wear exposure is linked to electrical degradation and loss of bioelectronic function. Cao et al. [17] and Wang et al. [18] tracked electrical changes during repeated rubbing or abrasion and assessed electrophysiological performance after wear. These measurements connect electrical durability with retained sensing performance, but do not continuously establish the complete progression from material damage to functional failure. A broader group demonstrates electrical stability and usable device function after rubbing, abrasion or washing, but does not determine how performance evolves as damage progressively accumulates [32,33,40,41,42,43]. Other studies show that material wear, electrical change and functional performance need not evolve in parallel [44,45,46,47]. The literature therefore supports tribological reliability as a functional concern across multiple bioelectronic modalities and material architectures, but complete mapping from wear exposure through material and electrical degradation to functional failure remains uncommon.

Cross-study comparison requires normalization of both tribological exposure and device response, but normalization cannot eliminate differences in test conditions. Where the necessary information is available, cumulative sliding distance should be reported alongside cycle number, normal load or contact pressure, counter-surface characteristics, sliding speed and environmental conditions. Electrical changes can be expressed relative to the initial resistance or impedance, while functional changes should be reported relative to the initial performance using a modality-specific metric, such as signal-to-noise ratio, calibrated sensitivity or analytical response. These measures allow degradation to be tracked on a common basis within a given test configuration. However, equal sliding distances or relative performance changes do not establish equivalent durability when the counter-surface, contact pressure or environmental conditions differ. Quantitative comparisons between independent studies should therefore be restricted to sufficiently matched exposure conditions and equivalent functional endpoints; otherwise, the results should be interpreted as evidence obtained under different test conditions rather than used to rank device durability.

## 5. Outlook—Toward Meaningful Evaluation of Tribological Reliability

A central challenge for the field is that tribological durability is currently demonstrated using tests that are difficult to compare. Devices may be reported to survive rubbing or abrasion for a given number of cycles, yet cycle count does not define the severity of the exposure. Future studies should therefore describe tribological loading in physical terms, including the counter-surface, normal load, sliding distance or amplitude, speed and environmental conditions, together with the criterion used to determine failure. This would allow durability claims to be interpreted in relation to the actual exposure rather than repetition alone.

The exposure itself should be selected according to the intended use of the device. A universal abrasion protocol is unlikely to represent the different contact conditions encountered by skin-mounted, exposed, textile-integrated and reusable systems. Testing should instead reproduce the dominant wear mode expected during use and, where appropriate, combine tribological loading with relevant deformation. Environmental conditions should follow the same application-specific principle. Humidity, simulated sweat or temperature should be incorporated when they represent credible conditions of use or are expected to materially alter the polymer or interface, rather than becoming mandatory additions to every durability test [6,11,34,35].

**Figure 2:**
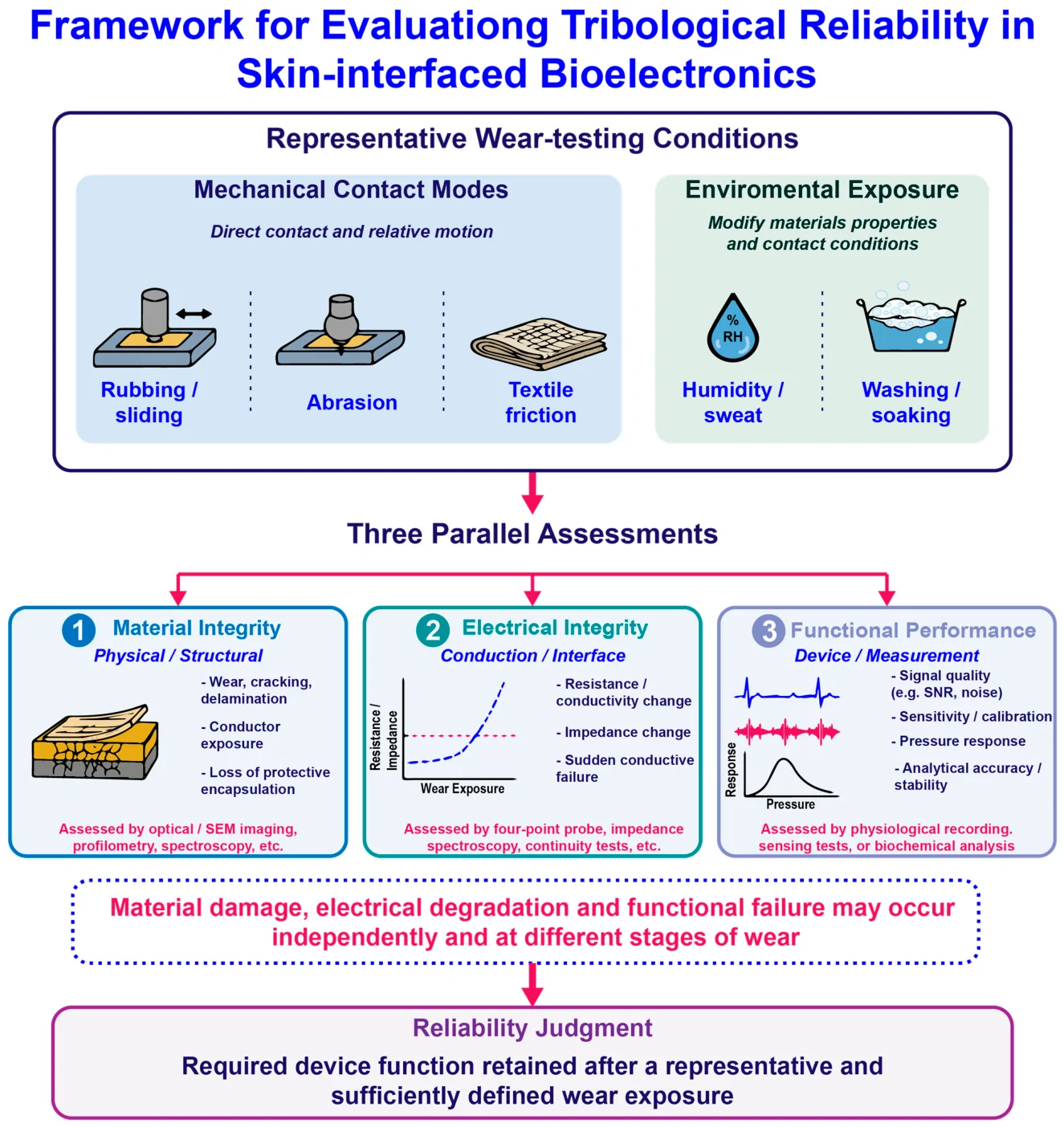
Application-relevant wear exposure followed by parallel assessment of material, electrical and functional outcomes, with failure potentially occurring independently at each level before an overall reliability judgement. Colors distinguish the three assessment pathways, arrows indicate the evaluation workflow, and dashed or dotted lines are used schematically to indicate thresholds or grouped stages rather than quantitative data.

The evaluation endpoint is equally important. Tribological testing should connect material, electrical and functional assessment rather than relying on any one of these outcomes alone (Figure 2). Morphological characterization can identify the location and progression of damage, while electrical and functional measurements establish whether that damage affects device operation. Measurements before and after exposure are essential, but measurements during progressive wear would be particularly valuable because they can identify when degradation begins and how it develops toward functional failure. Predictive modeling could complement experimental testing by identifying regions where contact loading is concentrated before substantial wear develops. Finite-element analysis has been used to examine interfacial stress and potential delamination in skin-mounted electronics under deformation [49], while simulations of polymer sliding contacts have been compared with experimentally measured wear profiles [50]. Extending these approaches to skin-interfaced polymer composites could help identify vulnerable conductive layers and buried interfaces, and guide the selection of contact locations and loading conditions for wear tests. Nevertheless, predictions of contact stress alone do not establish tribological lifetime or functional reliability. Models should be informed by measured material and interfacial properties and validated against observed damage, electrical changes and sensing performance.

A device should therefore be described as tribologically reliable only when it retains the performance required for its intended application after a representative and sufficiently defined wear exposure. This does not require a universal failure threshold. Acceptable changes in resistance, impedance, sensitivity, signal quality or analytical response depend on the function of the device. Standardization should consequently focus on a common framework for defining exposure, documenting degradation and establishing application-specific failure criteria. At minimum, studies should report the counter-surface, normal load, sliding distance or amplitude, speed, cycle number, relevant environmental conditions and failure criterion, together with material, electrical and functional outcomes where appropriate.

## 6. Conclusions

Tribological reliability is a distinct requirement for skin-interfaced bioelectronics that cannot be established through deformation tolerance or retained electrical continuity alone. The studies reviewed show that polymer-composite architecture and interfacial design influence how repeated contact produces material damage and whether that damage compromises electrical and sensing performance. Nevertheless, evidence tracing progressive wear through material degradation to predefined functional failure remains limited, while differences in testing conditions and performance endpoints restrict comparisons between studies. The proposed application-specific framework addresses these limitations by linking representative wear exposure to material, electrical and functional assessment, with failure criteria defined by the intended device function. Future studies should prioritize measurements at successive wear intervals and establish whether laboratory exposure reproduces the damage mechanisms encountered during use. This would provide a stronger basis for evaluating the long-term functional reliability of skin-interfaced polymer composites.

## Data Availability

No new data were created or analyzed in this study. Data sharing is not applicable to this article.

## References

[1] Cheng H., Du Y., Wang B., Mao Z., Xu H., Zhang L., Zhong Y., Jiang W., Wang L., Sui X. (2018). Flexible Cellulose-Based Thermoelectric Sponge towards Wearable Pressure Sensor and Energy Harvesting. Chem. Eng. J..

[2] Li T., Qi H., Zhao C., Li Z., Zhou W., Li G., Zhuo H., Zhai W. (2025). Robust Skin-Integrated Conductive Biogel for High-Fidelity Detection under Mechanical Stress. Nat. Commun..

[3] Zhou X., Kateb P., Fan J., Kim J., Lodygensky G.A., Amilhon B., Pasini D., Cicoira F. (2024). Conducting Polymer Films and Bioelectrodes Combining High Adhesion and Electro-Mechanical Self-Healing. J. Mater. Chem. C.

[4] Chang B., Luo J., Liu J., Zhang B., Zhu M., Li K., Li Y., Zhang Q., Shi G., Hou C. (2024). A High-Performance Composite Fiber with an Organohydrogel Sheath for Electrocardiogram Monitoring. J. Mater. Chem. C.

[5] Zhuo S., Tessier A., Arefi M., Zhang A., Williams C., Ameri S.K. (2024). Reusable Free-Standing Hydrogel Electronic Tattoo Sensors with Superior Performance. npj Flex. Electron..

[6] Liu Y., Cheng Y., Shi L., Wang R., Sun J. (2022). Breathable, Self-Adhesive Dry Electrodes for Stable Electrophysiological Signal Monitoring During Exercise. ACS Appl. Mater. Interfaces.

[7] Yi J., Gu Y., Yang J., Wang Z., Wang Y., Yan W., Sun Q., Zhou P., Xu Y., He X. (2025). Ultrathin and Permeable Silver Nanowires/Polyvinyl Alcohol Epidermal Electrode for Continuous Electrophysiological Monitoring. Mater. Horiz..

[8] Wang W., Xu G., Huang K., Ren C., Wu Y., Zhou Y., Cheng Z., Zhang S., Li H., Yang C. (2025). Cholinium-Based Eutectogel Electrode for High-Quality Dynamic EEG/ECG Monitoring Exceeding 48 Hours. npj Flex. Electron..

[9] Song J., Kim Y., Kang K., Lee S., Shin M., Son D. (2022). Stretchable and Self-Healable Graphene–Polymer Conductive Composite for Wearable EMG Sensor. Polymers.

[10] Yu S., Ji Z., Liu L., Huang Z., Luo Y., Wang H., Wangyuan R., Guo Z., Chen Z., Liao Q. (2025). A Hyperconformal Dual-Modal Metaskin for Well-Defined and High-Precision Contextual Interactions. Nat. Commun..

[11] Zhang L., Kumar K.S., He H., Cai C.J., He X., Gao H., Yue S., Li C., Seet R.C.-S., Ren H. (2020). Fully Organic Compliant Dry Electrodes Self-Adhesive to Skin for Long-Term Motion-Robust Epidermal Biopotential Monitoring. Nat. Commun..

[12] Ye H., Wu B., Sun S., Wu P. (2024). Self-Compliant Ionic Skin by Leveraging Hierarchical Hydrogen Bond Association. Nat. Commun..

[13] Somarathna U.S., Pathirana O.V., Wickramasinghe G.C., Alhendi M.A., Weerawarne D.L., Poliks M.D. (2026). Evaluation of Electromechanical Reliability of Flexible and Stretchable Electronics. Annu. Rev. Mater. Res..

[14] Tu H., Li X., Lin X., Lang C., Gao Y. (2023). Washable and Flexible Screen-Printed Ag/AgCl Electrode on Textiles for ECG Monitoring. Polymers.

[15] Zhao C., Park J., Root S.E., Bao Z. (2024). Skin-Inspired Soft Bioelectronic Materials, Devices and Systems. Nat. Rev. Bioeng..

[16] Kim H.J., Koo J.H., Lee S., Hyeon T., Kim D.-H. (2025). Materials Design and Integration Strategies for Soft Bioelectronics in Digital Healthcare. Nat. Rev. Mater..

[17] Cao J., Liu X., Qiu J., Yue Z., Li Y., Xu Q., Chen Y., Chen J., Cheng H., Xing G. (2024). Anti-Friction Gold-Based Stretchable Electronics Enabled by Interfacial Diffusion-Induced Cohesion. Nat. Commun..

[18] Wang Z., Shi P., Li Y., Liu T., Zhu B., Liu J., Deng Z., Wang Y., Zhao Y., Xiao Z. (2026). Abrasion-Resistant Wearable Skins Based on Bilayered Solid/Liquid Stretchable Conductors. Nat. Commun..

[19] Qaiser N., Suryaprabha T., Vetrano F., Hwang B., Elatab N. (2026). Mechanics and Bio-Interface Engineering in Flexible Biosensors for Continuous Health Monitoring. npj Flex. Electron..

[20] Dong F., Han C., Cai S.T., Lee J., Niu S. (2025). Skin-Integrated Wearable Electronics: A Dual-Interface Perspective. SmartSys.

[21] Yuk H., Wu J., Zhao X. (2022). Hydrogel Interfaces for Merging Humans and Machines. Nat. Rev. Mater..

[22] Ojstršek A., Jug L., Plohl O. (2022). A Review of Electro Conductive Textiles Utilizing the Dip-Coating Technique: Their Functionality, Durability and Sustainability. Polymers.

[23] Shi H.H., Pan Y., Xu L., Feng X., Wang W., Potluri P., Hu L., Hasan T., Huang Y.Y.S. (2023). Sustainable Electronic Textiles towards Scalable Commercialization. Nat. Mater..

[24] Song H., Liang Y., Zhang G., Long K., Shi K., Han X., Liu C., Qing Y. (2026). Superhydrophobic Wearable Strain Sensors: From Strategic Design to Robustness Paradigm. Nano-Micro Lett..

[25] Nabeel M., Jaber H., Taieh N.K., Li Y., Gou G., Kovács T. (2026). Reliability of Flexible Pressure Sensors under Sweat, Moisture, and Environmental Stress: Mechanisms, Materials, Packaging, and Data-Driven Mitigation. Sens. Actuators A Phys..

[26] Huang H., Cai X., Wan S., Sun L. (2026). Source-Level Motion Artifact Suppression in Wearable Electrodes: From Underlying Mechanisms to Advanced Strategies. Mater. Today Bio.

[27] Yin J., Wang S., Tat T., Chen J. (2024). Motion Artefact Management for Soft Bioelectronics. Nat. Rev. Bioeng..

[28] Tang W., Zhou Y., Chen S., Yu S., Yang Y., Lin J., Yin S., Ma Y., Hu B. (2021). Delamination-Resistant Imperceptible Bioelectrode for Robust Electrophysiological Signals Monitoring. ACS Mater. Lett..

[29] Kim J., Zhang G., Shi M., Suo Z. (2021). Fracture, Fatigue, and Friction of Polymers in Which Entanglements Greatly Outnumber Cross-Links. Science.

[30] Bayrak S., Paulkowski D., Stöckelhuber K.W., Staar B., Mayer B. (2020). A Comprehensive Study about the Role of Crosslink Density on the Tribological Behavior of DLC Coated Rubber. Materials.

[31] Le Gal A., Yang X., Klüppel M. (2005). Evaluation of Sliding Friction and Contact Mechanics of Elastomers Based on Dynamic-Mechanical Analysis. J. Chem. Phys..

[32] Chen Y., Liu Z., Wang Z., Yi Y., Yan C., Xu W., Zhou F., Gao Y., Zhou Q., Zhang C. (2024). Bioinspired Robust Gas-Permeable On-Skin Electronics: Armor-Designed Nanoporous Flash Graphene Assembly Enhancing Mechanical Resilience. Adv. Sci..

[33] Cao C., Su H., Ai L., Lv D., Gu J., Li R., Li D., Zhang W., Ge M., Yao X. (2024). Highly Stable Liquid Metal-Based Electronic Textiles by Adaptive Interfacial Interactions. Adv. Funct. Mater..

[34] Derler S., Gerhardt L.-C. (2012). Tribology of Skin: Review and Analysis of Experimental Results for the Friction Coefficient of Human Skin. Tribol. Lett..

[35] Gerhardt L.-C., Strässle V., Lenz A., Spencer N.D., Derler S. (2008). Influence of Epidermal Hydration on the Friction of Human Skin against Textiles. J. R. Soc. Interface.

[36] Tian Y., Wang Z., Cao S., Liu D., Zhang Y., Chen C., Jiang Z., Ma J., Wang Y. (2024). Connective Tissue Inspired Elastomer-Based Hydrogel for Artificial Skin via Radiation-Indued Penetrating Polymerization. Nat. Commun..

[37] Macron J., Gerratt A.P., Lacour S.P. (2019). Thin Hydrogel–Elastomer Multilayer Encapsulation for Soft Electronics. Adv. Mater. Technol..

[38] Wang Z., Wang Y., Yang J., Zhou P., Yan W., Lin S., Zhang Y., Sun Q., Xu Y., Ji Z. (2025). Sub-400 Nanometer-Thick Skin and Environment Adaptable Organohydrogel Nanofilm Epidermal Electrode. Nat. Commun..

[39] Pradeep Raja C., Satyanarayana V.S.V., Venkata Siva Teja P., Venkata Suresh B., Sridevi G., Pandipati S., Mensah R.A., Karthik Babu N.B. (2026). An In-Depth Study on Tribological Behaviour of Polymers and Polymer Composites: State-of-the-Art. Front. Mater..

[40] Ding L., Hang C., Yang S., Qi J., Dong R., Zhang Y., Sun H., Jiang X. (2022). *In Situ* Deposition of Skin-Adhesive Liquid Metal Particles with Robust Wear Resistance for Epidermal Electronics. Nano Lett..

[41] Jeong S., Heo S., Kang M., Kim H.-J. (2020). Mechanical Durability Enhancement of Gold-Nanosheet Stretchable Electrodes for Wearable Human Bio-Signal Detection. Mater. Des..

[42] Xu Z., Guo H., Cai J., Wang F., Li J., Tang Q., Yu J., Ding B., Li Z. (2026). Breathable and Reusable Fabric Epidermal Electrodes for Personal Health Monitoring. Nano Energy.

[43] Zhang M., Wang W., Yuan Y., Wen D., Farghaly M., Zhang C., Yan S., Quan Y., Xiao X. (2026). Breathable and Mechanically Robust Textile Electrode Based on Grooved Silver-Plated Fibers for Reliable Long-Term ECG Monitoring. ACS Appl. Polym. Mater..

[44] Fang J., Jia Y., Liao Z., Qi B., Huang T. (2025). A Mechanical–Electrochemical Dual-Model E-Skin for the Monitoring of Cardiovascular Healthcare. Biosensors.

[45] Jiang J., Song X., Qi Y., Tao X., Zheng Z., Huang Q. (2025). Skin-Inspired, Permeable, Structure-Gradient Fiber Mats for Pressure Sensing in Rehabilitation Assistance. Adv. Fiber Mater..

[46] Lee S., Kim J.S., Wang Y., Tagawa Y., Wang W., Sun L., Liang X., Goni Nayeem M.O., Yokota T., Fukuda K. (2025). An Ultrasoft Nanomesh Strain Sensor with Extreme Mechanical Durability against Friction for On-Skin Applications. Device.

[47] Luo D., Liu L., Liu Y., Li J., Li Z., Sun H., Peng P., Qiu Y., Liu Y., Liu H. (2025). Rubbing Resistant and Rapid Response Multifunctional Sweat Sensor Based on Core-Sheath Structure Yarns. Chem. Eng. J..

[48] Li G., Wang S., Duan Y.Y. (2017). Towards Gel-Free Electrodes: A Systematic Study of Electrode-Skin Impedance. Sens. Actuators B Chem..

[49] Choi G., Kim J., Kim H., Bae H., Kim B., Lee H.J., Jang H., Seong M., Tawfik S.M., Kim J.J. (2025). Motion-Adaptive Tessellated Skin Patches With Switchable Adhesion for Wearable Electronics. Adv. Mater..

[50] Awwad K.Y.E., Fallahnezhad K., Yousif B.F., Mostafa A., Alajarmeh O., Shalwan A., Zeng X. (2024). Finite Element Analysis and Experimental Validation of Polymer–Metal Contacts in Block-on-Ring Configuration. Friction.

